# Triple Trouble: Early Clues in the Diagnosis of Allgrove Syndrome: A Case Report

**DOI:** 10.1155/crie/3771629

**Published:** 2026-05-16

**Authors:** Titilope Majiyagbe, Anitha Kumaran, Lawrence Gnanaraj, Claire Wood

**Affiliations:** ^1^ Paediatric Endocrinology Department, Newcastle Upon Tyne Hospital NHS Foundation Trust, Newcastle, UK, ncl.ac.uk; ^2^ Paediatric Endocrinology Department, University Hospital Southampton, Hampshire, UK, nhs.uk; ^3^ Sunderland Eye Infirmary, Sunderland and South Tyneside NHS Foundation Trust, Sunderland, UK, nhs.uk; ^4^ Translational and Clinical Research Institute, Newcastle University, Newcastle, UK, ncl.ac.uk

**Keywords:** achalasia, adrenal insufficiency, alacrima, Allgrove

## Abstract

**Introduction:**

Allgrove syndrome (also known as Triple A syndrome) is an inherited autosomal recessive condition, characterised by achalasia, alacrima and adrenal insufficiency. Its rarity and high phenotypic heterogeneity mean that it often poses a diagnostic challenge.

**Case Presentation:**

We discuss the case of a previously well 5‐year‐old who presented with adrenal crisis. The R150 genetic panel showed a mutation in the achalasia‐addisonianism‐alacrima syndrome (AAAS) gene, confirming a diagnosis of Allgrove syndrome. It was noted in retrospect that his earliest presenting feature was alacrima, which was noted by parents in the neonatal period but not investigated. He was started on glucocorticoid replacement and is under regular paediatric endocrine outpatient follow‐up.

**Conclusion:**

If our patient’s alacrima had come to medical attention and Allgrove syndrome been diagnosed earlier, then the subsequent adrenal crisis could probably have been avoided, as regular monitoring of the adrenal axis would have been undertaken.

**Summary**

 Established Facts

• Already known facts
° Allgrove syndrome is a rare autosomal recessive disorder caused by mutations in the achalasia‐addisonianism‐alacrima syndrome (AAAS) gene and is classically associated with the triad of alacrima, achalasia, and adrenal insufficiency.
° Adrenal crisis is a common cause of morbidity and mortality in undiagnosed or late‐diagnosed cases.
 Novel Insights
• New information
° The patient presented with adrenal crisis earlier than typically reported, suggesting that the onset of adrenal insufficiency in Allgrove syndrome may occur at a younger age than classically described.
° There was clinical overlap between eczema and hyperpigmentation, illustrating how ethnicity and skin tone may complicate recognition of adrenal‐related skin signs.
° Optic nerve dysfunction was identified through visual evoked potential (VEP) test before overt visual symptoms, suggesting that subclinical neurological involvement may be an early feature of the disease, reinforcing the need for early ophthalmological surveillance.

## 1. Introduction

Allgrove syndrome, also known as Triple A syndrome, is characterised by a triad of alacrima, achalasia, and adrenal insufficiency. It is an autosomal recessive condition, caused by mutation of the achalasia‐addisonianism‐alacrima syndrome (AAAS) gene on chromosome 12q13, which encodes the Alacrima‐Achalasia‐Adrenal Insufficiency‐Neurologic disorder (ALADIN) protein [1]. Allgrove syndrome has an estimated prevalence of 1 in 1 million, although the actual burden may be much higher as a result of missed diagnoses [2] because there is considerable variability in the timing to presentation and severity of the associated features [3]. It is a progressive disorder and depending on the presenting features, diagnosis may take years [4].

## 2. Case Report/Case Presentation

### 2.1. Patient Information

A 5‐year‐old male attended the paediatric Emergency Department (ED) at Portsmouth whilst visiting his father who lived there. He presented with an acute onset of generalised seizures following a short history of viral upper respiratory tract infection, lethargy and reduced oral intake. His father reported that the patient’s appetite had been poor throughout the day, which was initially attributed to the intercurrent illness. On the evening prior to the seizure episode, the patient consumed only a small amount of food (a few chicken nuggets) for dinner and had minimal intake earlier in the day.

The patient is of Afro‐Caribbean ethnicity with no family history of genetic disorders or parental consanguinity. He was born at term with a good weight. He had surgery for a left trigger thumb in the newborn period and had hypopigmented patches thought to be pityriasis alba secondary to eczema, which was being managed with steroid cream (detailed further below).

### 2.2. Clinical Findings

Dad noticed in the early hours of the morning (~4:30 AM) that he was hot and having ‘the shakes’. He also observed that he was unresponsive to voice with upward rolling of the eyes. He had two more episodes: one before dad drove him to the ED and a further 3‐min generalised tonic‐clonic convulsion on arrival in the ED. At triage he had a blood glucose of 1.1 mmol/L. The patient was pyrexial with a recorded temperature of 38.2°C but showed no evidence of cardiovascular compromise. Initial blood investigations demonstrated a serum sodium level of 135 mmol/L and a potassium level of 4.0 mmol/L. Urea and creatinine were mildly elevated.

Based on the clinical history of intercurrent illness with reduced oral intake and the laboratory findings, the initial working diagnosis was severe ketotic hypoglycaemia secondary to illness and poor oral intake.

### 2.3. Timeline

The patient was reviewed by the endocrine team after initial stabilisation of hypoglycaemia and pending the results of the hypoglycaemic screen.

On direct questioning, it was ascertained that the patient had cried with no tears since birth; this information was not forthcoming from the parents (as they had no expectation that it could be associated with his current presentation) or local team and was only obtained on specialist review by the endocrine team.

There was also a history of skin hyperpigmentation of his testes over a few months leading to presentation. The family had assumed this was a natural colour change consistent with his ethnicity. Neither feature had been reported or investigated prior to this episode. The patient had previously seen dermatology in infancy with patches of hyperpigmentation and had continued to have patches of depigmentation on his face. He had previously been reviewed by a dermatologist who attributed it to pityriasis alba, secondary to eczema and advised treatment with topical steroid. The skin pigmentation had improved since starting hydrocortisone.

On examination, he had no dysmorphic features. He was observed to have skin hyperpigmentation of the lower trunk, abdomen, testes and axilla with sparing of the face and upper trunk. Three hyperpigmented birthmarks were noted on his abdomen in the midline, right lower quadrant and right lateral hip. The nail beds, palmar creases and mucosal surfaces were normal.

Ophthalmic examination confirmed an unaided visual acuity of 0.5 LogMAR bilaterally, which improved to 0.2 LogMAR with spectacle correction. Colour vision testing using the Pseudoisochromatic Ishihara chart demonstrated a generalised inability to distinguish numbers, indicating a significant red‐green deficiency.

His anterior segment and pupillary examination were unremarkable with no evidence of relative afferent pupillary defect (RAPD). His fundus examination demonstrated bilateral temporal optic disc pallor.

The remainder of the systemic examination was unremarkable.

### 2.4. Therapeutic Intervention

The patient was given 2 mL/kg of 10% glucose and then a further 2 mL/kg bolus to treat refractory hypoglycaemia. He was given a 50 mg bolus of IV hydrocortisone which was then repeated every 6 h for 24 h. Glucose concentrations were maintained with continuous 5% glucose infusion. Full blood count showed a mildly elevated white cell count with a neutrophilia. Chest X‐ray showed right‐sided shadowing and antibiotics were commenced.

### 2.5. Diagnostic Assessment

Laboratory testing confirmed hypoglycaemia with glucose levels of 1.1 mmol/L and a cortisol level of 43 nmol/L. Other components of the hypoglycaemia screen were normal, including ammonia 33 μmol/L, lactate 1.5 mmol/L, growth hormone 14.3 μg/L, and insulin 0.2 mIU/mL with undetectable levels of C‐peptide. Thyroid function testing was normal with negative thyroglobulin and thyroid peroxidase antibodies. Ultrasound scan of the abdomen showed no evidence of adrenal haemorrhage.

He had a suboptimal response on short Synacthen test: baseline cortisol of 23 nmol/L, 30‐min value of 28 nmol/L and cortisol of 24 nmol/L at 60 min. His ACTH was raised at 2280 pg/mL, consistent with a diagnosis of primary adrenal insufficiency (PAI). Normal androgen levels, urinary steroid profile, plasma amino acids and very long‐chain fatty acid profile were subsequently returned. Coeliac screen and adrenal antibodies were also negative.

Following diagnosis of adrenal insufficiency, the patient was commenced on replacement glucocorticoid therapy: hydrocortisone 10.5 mg/m^2^/day (5 mg at 06:30, 2.5 mg at 14:00 and 1.5 mg at 20:00).

Serum electrolytes showed no evidence of salt wasting, and blood pressure remained within the normal range. There was no hyponatraemia or hyperkalaemia, and therefore no clear clinical indication for immediate mineralocorticoid replacement. However, given the patient’s age, PAI was considered more likely than secondary causes. In view of the possibility of subclinical mineralocorticoid deficiency, the patient was also commenced on fludrocortisone at a dose of 50 mcg once daily, while awaiting completion of the adrenal work‐up. At the time of initiation, plasma renin and aldosterone levels were not yet available.

The intention was to review and discontinue fludrocortisone if subsequent investigations indicated that mineralocorticoid replacement was not required. Unfortunately, the initial sample for renin and aldosterone measurement was reported as haemolysed, necessitating repeat testing. Subsequent measurements demonstrated normal renin and aldosterone levels.

A presumptive diagnosis of Allgrove syndrome was made, based on the alacrima and adrenal insufficiency and the R150 panel was requested to investigate genetic causes of PAI.

Schirmer’s test was not performed, as this is not routinely indicated in asymptomatic young patients.

Optical coherence tomography (OCT) revealed retinal nerve fibre layer thinning in both eyes, more pronounced in the left eye, along with significant loss of the macular ganglion cell layer. Pattern‐reversal visual evoked potential (VEP) test demonstrated poorly formed and delayed responses in both eyes, suggestive of optic pathway dysfunction.

### 2.6. Follow‐Up and Outcomes

The patient was discharged home on a steroid regimen with basic training given to dad regarding sick day rules and emergency hydrocortisone injections. The local clinicians were made aware of the patient and family was linked with the local team for follow up and subsequent training, including of mum (with whom the patient normally resides) and the school/childminder.

The R150 panel confirmed compound heterozygous mutations for two pathogenic variants in the AAAS gene, and a diagnosis of Allgrove syndrome was made. The fludrocortisone was discontinued following genetic confirmation of Allgrove syndrome, and the family chose to switch to Alkindi hydrocortisone granules for his regular glucocorticoid dosing for ease.

The patient has had VEP testing which is suggestive of bilateral optic nerve dysfunction. He will need further visual assessment when he is older and better able to tolerate corneal electrodes. A recent barium swallow to assess oesophageal motility was normal with no features of achalasia seen. The school is aware of his diagnosis and the potential need for extra support.

## 3. Discussion

Allgrove (or Triple A) syndrome is a disease characterised by the presence of alacrima, achalasia and adrenal insufficiency. It was first described in 1978 and is an autosomal recessive condition, caused by a mutation in the AAAS gene. The complete triad is present in around 70% of patients and many patients also have autonomic nervous system dysfunction; hence, it is sometimes referred to as 4A syndrome [5]

Our patient presented with alacrima as the earliest feature of his subsequent diagnosis. This is present in around 90% of patients with Allgrove syndrome and is often the first presenting and most consistent feature [6]. However, consistent with other case series, its significance in our patient was not recognised and medical assistance was not sought until other symptoms developed [7], and he presented acutely in adrenal crisis.

Alacrima in Allgrove syndrome is thought to be due to a deterioration of the autonomic innervation of the lacrimal gland and a resultant lacrimal gland atrophy [8]. In addition to alacrima, Allgrove syndrome can present with other ophthalmic manifestations, including keratoconjunctivitis, abnormalities of the pupils, including sluggish and tonic pupils and optic atrophy [8, 9]. Our patient also demonstrated bilateral temporal optic disc pallor with poorly formed and/or delayed amplitude in both eyes on VEP. This suggests axonal degeneration and may represent an additional sign of nerve degeneration [9]. Monitoring by an ophthalmologist is recommended, at least yearly, and the use of artificial tears/lubricants can prevent corneal ulceration. Our patient; however, was asymptomatic with normal ocular surface examination; no topical treatment was indicated.

Adrenal crisis with associated hypoglycaemia and/or hypotension, as in our patient, remains the leading cause of mortality in Allgrove syndrome [2]. Historically, adrenal insufficiency was thought to manifest later in the first decade of life, and in some cases not until adolescence or adulthood [10]. However, with increasing documentation through case reports and case series, it is now evident that the age of presentation is highly variable.

Our patient presented in adrenal crisis shortly after turning 5 years of age, which is earlier than that reported in most case series [5]. There is, however, a case report documenting the complete triad of Allgrove syndrome as early as 6 months of age [11].

These findings underscore the expanding phenotypic spectrum and the need for heightened clinical suspicion across all paediatric age groups.

The AAAS gene product is the nuclear pore complex protein ALADIN, which plays a role in redox homeostasis in human adrenal cells and inhibits steroidogenesis [12]. Deficiency of this protein causes increased sensitivity of adrenal and neuronal fibroblasts to oxidative stressors [12]. Progressive adrenal cell atrophy and ACTH resistance occur [7] and are characterised by the presence of low cortisol levels and markedly elevated serum ACTH concentrations [13].

This insidious onset may explain why symptoms of adrenal insufficiency frequently do not occur in Allgrove syndrome until the patient has a decompensating illness/event which precipitates an adrenal crisis. It is also likely that at least some of the skin changes in our patient, which were thought to be secondary to eczema, were in fact hyperpigmentation in other areas, associated with ACTH resistance. The ethnicity of our patient may have made it more challenging to establish the presence of skin hyperpigmentation.

Most patients with Allgrove syndrome have isolated glucocorticoid deficiency; however, mineralocorticoid deficiency has occasionally been reported [14], so it is important to determine this to ensure appropriate treatment. One hypothesis for the relative conservation of the zona glomerulosa could be reduced production of reactive oxygen species during aldosterone biosynthesis [14]. Studies have also detected low DHEAS levels, suggesting progressive atrophy of the zona reticularis as well as the impaired steroidogenesis in the zona fasciculata [14].

Achalasia of the cardia occurs in about 75% of all cases, with a variable age at onset ranging from infancy to 16 years [15]. It can be insidious in onset, and the presenting symptoms often include vomiting, dysphagia, weight loss and severe malnutrition [16]. Dysphagia may be present for years before a diagnosis of achalasia is made and may present as gastroesophageal reflux [7].

Neurological symptoms involving the central, peripheral and autonomic nervous systems are well documented in the literature [17]. Reported manifestations include hyperreflexia, extensor plantar responses, altered muscle tone, muscle weakness and wasting, ataxia, dysarthria, sensory impairment and optic atrophy [13]. The pathophysiology underlying these neurological features remains incompletely understood [3]. Neurological symptoms are typically reported to manifest during the second decade of life or in adulthood; however, several reports indicate that they may occur earlier and, in some cases, may even represent presenting features in childhood [3, 5]. The age of onset is therefore variable, and neurological abnormalities may precede the classical components of the syndrome.

Neuroimaging studies have occasionally demonstrated focal demyelination in regions such as the medulla and inferior cerebellar peduncle, although findings are generally non‐specific and no consistent pattern of brain parenchymal abnormalities has been reported in Allgrove syndrome [18].

The presence of optic nerve atrophy in our patient likely represents neuro‐ophthalmological involvement within the recognised disease spectrum.

Whilst the presence of two of the three of these features strongly suggested the diagnosis of Allgrove syndrome at the time of presentation in adrenal crisis in our patient, the recognition of the clinical syndrome is a challenge at the onset of the disease, when only one presenting symptom is typically observed [3]. This case highlights the importance of thorough history taking and examination to recognise rarer forms of PAI and a systematic approach to differential diagnosis, especially when definitive diagnosis has not been made.

Genetic testing is often performed to confirm the diagnosis. Prompt genetic diagnosis enables genetic counselling prior to future pregnancies and thus pre‐natal diagnosis if appropriate and the potential identification of early symptoms in siblings.

## 4. Conclusion

While Allgrove syndrome is a rare condition, it should be considered in every child who presents with alacrima [3] and in those who present with adrenal insufficiency/crisis. If our patient’s alacrima had come to medical attention and Allgrove syndrome been diagnosed earlier, then the subsequent adrenal crisis could probably have been avoided, as regular monitoring of the adrenal axis would have been undertaken. Further considerations to optimise management of Allgrove syndrome and prevent adrenal crisis and its associated morbidity could include developing a surveillance pathway for monitoring of infants/children with alacrima. Although our patient does not currently have any features of achalasia, it will be prudent to ask at clinic appointments if there are any new signs/symptoms of dysmotility and make a prompt referral for further investigation and management if required, as well as providing appropriate support for any neurological sequelae as they arise.

## Author Contributions

Anitha Kumaran saw the patient at initial presentation and Claire Wood is responsible for ongoing management. Lawrence Gnanaraj continues to monitor patient’s visual function. Titilope Majiyagbe has also clinically reviewed the patient and wrote the first draft of the manuscript.

## Funding

No relevant funding sources.

## Disclosure

All authors have been involved in reviewing the drafts and in the approval of the submitted manuscript.

## Ethics Statement

Written informed consent was obtained from the parent for publication of the details of their son’s medical case.

## Conflicts of Interest

The authors declare no conflicts of interest.

## Data Availability

Data can be made available upon written request to the corresponding author.
